# Supplementary material for: Deploying deep learning models on unseen medical imaging using adversarial domain adaptation
Source: PLoS One. 2022 Oct 14;17(10):e0273262. doi: 10.1371/journal.pone.0273262 (PMC9565422; doi:10.1371/journal.pone.0273262)
Supplement: S1 File — (PDF) [file pone.0273262.s001.pdf]

SUPPLEMENTAL TABLES

Supplemental Table 1. Characteristics of the digits datasets.

	MNIST	MNISTM	USPS	P-value
Images, n	70,000	70,000	9,298	
Digits, n (%)				
Zero	6,903 (9.9)	6,903 (9.9)	1,553 (16.7)	<0.001
One	7,877 (11.3)	7,877 (11.3)	1,269 (13.6)	<0.001
Two	6,990 (10.0)	6,990 (10.0)	929 (10.0)	0.998
Three	7,141 (10.2)	7,141 (10.2)	824 (8.9)	<0.001
Four	6,824 (9.7)	6,824 (9.7)	852 (9.2)	0.182
Five	6,313 (9.0)	6,313 (9.0)	716 (7.7)	<0.001
Six	6,876 (9.8)	6,876 (9.8)	834 (9.0)	0.027
Seven	7,293 (10.4)	7,293 (10.4)	792 (8.5)	<0.001
Eight	6,825 (9.8)	6,825 (9.8)	708 (7.6)	<0.001
Nine	6,958 (9.9)	6,958 (9.9)	821 (8.8)	0.002

Supplemental Table 2. Characteristics of the chest x-ray datasets.

	Beth Israel	NIH	San Juan	Stanford	P-value
Radiographs, n	371,920	112,120	160,861	223,648	
AP/PA, n (%)	250,044 (67.3)	112,120 (100.0)	63,848 (39.7)	191,229 (85.5)	<0.001

Patients, n	65,088	30,805	67,625	64,740	
Patient demographics					
Age, mean (SD)	-	46.1 (16.7)	-	60.3 (18.6)	<0.001
Female, n (%)	-	14,175 (46.0)	34,986 (51.7)	28,822 (44.5)	<0.001
Opacity, n (%)	118,452 (47.4)	39,458 (35.2)	11,002 (18.9)	137,675 (72.0)	<0.001

Supplemental Table 3. Results of baseline bespoke models trained on chest x-rays from a single hospital site and evaluated on chest x-rays from target hospital sites.

Train Site (No. Images)	Comparison Type	Test Site (No. Images)	Accuracy (SD)	AUC (SD)	Sensitivity (SD)	Specificity (SD)	PPV (SD)	NPV (SD)
Beth Israel (N = 200,313)	Internal	Beth Israel (N = 24,780)	0.746 (0.013)	**0.819 (0.013)**	0.703 (0.019)	0.797 (0.019)	0.806 (0.018)	0.691 (0.019)
	External	NIH (N = 11,517)	0.654 (0.015)	0.694 (0.017)	0.515 (0.024)	0.760 (0.018)	0.618 (0.025)	0.674 (0.019)
	External	San Juan (N = 5,974)	**0.808 (0.012)**	0.729 (0.019)	0.556 (0.052)	**0.834 (0.012)**	0.255 (0.031)	**0.948 (0.008)**
	External	Stanford (N = 19,386)	0.771 (0.013)	0.779 (0.017)	**0.840 (0.013)**	0.585 (0.031)	**0.847 (0.014)**	0.571 (0.029)
NIH (N = 89,722)	Internal	NIH (N = 11,517)	0.665 (0.015)	0.713 (0.017)	0.528 (0.024)	0.763 (0.018)	0.616 (0.026)	0.692 (0.018)
	External	Beth Israel (N = 24,780)	0.675 (0.015)	**0.768 (0.015)**	0.612 (0.019)	0.806 (0.022)	0.867 (0.015)	0.500 (0.022)
	External	San Juan (N = 5,974)	**0.787 (0.013)**	0.699 (0.020)	0.455 (0.047)	**0.832 (0.013)**	0.267 (0.031)	**0.919 (0.010)**
	External	Stanford (N = 19,386)	0.747 (0.013)	0.718 (0.018)	**0.796 (0.014)**	0.551 (0.035)	**0.876 (0.012)**	0.405 (0.030)
San Juan (N = 46,495)	Internal	San Juan (N = 5,974)	0.685 (0.015)	**0.792 (0.018)**	0.368 (0.024)	**0.922 (0.011)**	0.779 (0.029)	**0.661 (0.017)**
	External	Beth Israel (N = 24,780)	0.560 (0.016)	0.675 (0.017)	0.520 (0.017)	0.882 (0.032)	0.973 (0.008)	0.184 (0.017)
	External	NIH (N = 11,517)	0.526 (0.016)	0.679 (0.018)	0.421 (0.018)	0.814 (0.024)	0.861 (0.018)	0.339 (0.019)
	External	Stanford (N = 19,386)	**0.740 (0.013)**	0.664 (0.020)	**0.741 (0.014)**	0.700 (0.079)	**0.986 (0.004)**	0.088 (0.017)

Stanford (N = 152,579)	Internal	Stanford (N = 19,386)	0.737 (0.014)	**0.800 (0.016)**	**0.872 (0.013)**	0.515 (0.026)	**0.748 (0.016)**	0.708 (0.027)
	External	Beth Israel (N = 24,780)	0.722 (0.014)	0.797 (0.014)	0.741 (0.021)	0.708 (0.019)	0.639 (0.022)	0.797 (0.017)
	External	NIH (N = 11,517)	0.672 (0.015)	0.676 (0.018)	0.553 (0.029)	0.721 (0.017)	0.445 (0.026)	0.799 (0.016)
	External	San Juan (N = 5,974)	**0.805 (0.013)**	0.694 (0.021)	0.671 (0.103)	**0.808 (0.013)**	0.072 (0.018)	**0.991 (0.003)**

Supplemental Table 4. Results of adapted models trained on chest x-rays from a single hospital site and evaluated on chest x-rays from target hospital sites. Yellow shading indicates baseline performance on internal validation. Green shading represents improvements on baseline performance via adaptation.

Train Site (No. Images)	Comparison Type	Test Site (No. Images)	Accuracy (SD)	AUC (SD)	Sensitivity (SD)	Specificity (SD)	PPV (SD)	NPV (SD)
Beth Israel (N = 200,313)	Internal	Beth Israel (N = 24,780)	0.746 (0.013)	0.819 (0.013)	0.703 (0.019)	0.797 (0.019)	0.806 (0.018)	0.691 (0.019)
	External	NIH (N = 11,517)	0.683 (0.015)	0.701 (0.018)	0.568 (0.029)	0.734 (0.017)	0.485 (0.027)	0.794 (0.016)
	External	San Juan (N = 5,974)	0.810 (0.012)	0.717 (0.021)	0.673 (0.083)	0.814 (0.012)	0.113 (0.023)	0.986 (0.004)
	External	Stanford (N = 19,386)	0.736 (0.014)	0.788 (0.017)	0.867 (0.013)	0.514 (0.026)	0.752 (0.016)	0.695 (0.029)
NIH (N = 89,722)	Internal	NIH (N = 11,517)	0.665 (0.015)	0.713 (0.017)	0.528 (0.024)	0.763 (0.018)	0.616 (0.026)	0.692 (0.018)
	External	Beth Israel (N = 24,780)	0.708 (0.014)	0.773 (0.015)	0.667 (0.020)	0.757 (0.021)	0.770 (0.019)	0.652 (0.020)
	External	San Juan (N = 5,974)	0.780 (0.013)	0.691 (0.021)	0.435 (0.042)	0.836 (0.013)	0.302 (0.032)	0.901 (0.010)
	External	Stanford (N = 19,386)	0.738 (0.014)	0.753 (0.018)	0.836 (0.014)	0.519 (0.029)	0.796 (0.015)	0.585 (0.030)
San Juan (N = 46,495)	Internal	San Juan (N = 5,974)	0.685 (0.015)	0.792 (0.018)	0.368 (0.024)	0.922 (0.011)	0.779 (0.029)	0.661 (0.017)
	External	Beth Israel (N = 24,780)	0.663 (0.015)	0.740 (0.016)	0.697 (0.025)	0.644 (0.019)	0.517 (0.023)	0.796 (0.018)
	External	NIH (N = 11,517)	0.667 (0.016)	0.692 (0.018)	0.537 (0.027)	0.735 (0.018)	0.516 (0.026)	0.751 (0.017)
	External	Stanford (N = 19,386)	0.509 (0.015)	0.718 (0.018)	0.867 (0.018)	0.340 (0.017)	0.382 (0.018)	0.844 (0.022)

Stanford (N = 152,579)	Internal	Stanford (N = 19,386)	0.737 (0.014)	0.800 (0.016)	0.872 (0.013)	0.515 (0.026)	0.748 (0.016)	0.708 (0.027)
	External	Beth Israel (N = 24,780)	0.725 (0.014)	0.801 (0.014)	0.738 (0.021)	0.716 (0.020)	0.655 (0.022)	0.789 (0.017)
	External	NIH (N = 11,517)	0.683 (0.015)	0.699 (0.017)	0.600 (0.035)	0.704 (0.016)	0.343 (0.025)	0.872 (0.013)
	External	San Juan (N = 5,974)	0.802 (0.013)	0.696 (0.020)	0.642 (0.136)	0.804 (0.013)	0.042 (0.014)	0.994 (0.003)

Supplemental Table 5. Results of baseline global models trained on incremental amounts of handwritten digits from all available datasets and evaluated on the global test set and dataset-specific test sets.

Train Site (No. Images)	Comparison Type	Test Site (No. Images)	Accuracy (SD)	AUC (SD)
MNIST + MNISTM + USPS 0.1% (N = 128)	All Datasets	MNIST + MNISTM + USPS (N = 22,007)	0.492 (0.016)	0.834 (0.008)
	Individual Dataset	MNIST (N = 10,000)	**0.630 (0.015)**	**0.931 (0.004)**
	Individual Dataset	MNISTM (N = 10,000)	0.384 (0.016)	0.719 (0.011)
	Individual Dataset	USPS (N = 2,007)	0.438 (0.016)	0.834 (0.007)
MNIST + MNISTM + USPS 1% (N = 1,273)	All Datasets	MNIST + MNISTM + USPS (N = 22,007)	0.798 (0.013)	0.973 (0.003)
	Individual Dataset	MNIST (N = 10,000)	**0.925 (0.008)**	**0.995 (0.001)**
	Individual Dataset	MNISTM (N = 10,000)	0.720 (0.014)	0.957 (0.004)
	Individual Dataset	USPS (N = 2,007)	0.743 (0.013)	0.960 (0.003)
MNIST + MNISTM + USPS 5% (N = 6,365)	All Datasets	MNIST + MNISTM + USPS (N = 22,007)	0.913 (0.009)	0.993 (0.001)
	Individual Dataset	MNIST (N = 10,000)	**0.978 (0.005)**	**0.999 (0.00)**
	Individual Dataset	MNISTM (N = 10,000)	0.871 (0.010)	0.988 (0.002)
	Individual Dataset	USPS (N = 2,007)	0.893 (0.010)	0.991 (0.002)
	All Datasets	MNIST + MNISTM + USPS (N = 22,007)	0.933 (0.008)	0.996 (0.001)

MNIST + MNISTM + USPS 10% (N = 12,730)	Individual Dataset	MNIST (N = 10,000)	**0.978 (0.005)**	**1.00 (0.00)**
	Individual Dataset	MNISTM (N = 10,000)	0.905 (0.009)	0.994 (0.001)
	Individual Dataset	USPS (N = 2,007)	0.942 (0.007)	0.994 (0.002)
MNIST + MNISTM + USPS 20% (N = 25,459)	All Datasets	MNIST + MNISTM + USPS (N = 22,007)	0.938 (0.008)	0.997 (0.001)
	Individual Dataset	MNIST (N = 10,000)	**0.986 (0.004)**	**1.00 (0.00)**
	Individual Dataset	MNISTM (N = 10,000)	0.921 (0.008)	0.997 (0.001)
	Individual Dataset	USPS (N = 2,007)	0.930 (0.008)	0.996 (0.001)
MNIST + MNISTM + USPS 40% (N = 50,917)	All Datasets	MNIST + MNISTM + USPS (N = 22,007)	0.954 (0.007)	0.998 (0.001)
	Individual Dataset	MNIST (N = 10,000)	**0.988 (0.003)**	**1.00 (0.00)**
	Individual Dataset	MNISTM (N = 10,000)	0.939 (0.008)	0.998 (0.00)
	Individual Dataset	USPS (N = 2,007)	0.951 (0.007)	0.997 (0.001)
MNIST + MNISTM + USPS 60% (N = 76,375)	All Datasets	MNIST + MNISTM + USPS (N = 22,007)	0.959 (0.006)	0.998 (0.001)
	Individual Dataset	MNIST (N = 10,000)	**0.991 (0.003)**	**1.00 (0.00)**
	Individual Dataset	MNISTM (N = 10,000)	0.946 (0.007)	0.999 (0.00)
	Individual Dataset	USPS (N = 2,007)	0.948 (0.007)	0.997 (0.001)
MNIST + MNISTM + USPS 80% (N = 101,833)	All Datasets	MNIST + MNISTM + USPS (N = 22,007)	0.964 (0.006)	0.998 (0.001)
	Individual Dataset	MNIST (N = 10,000)	**0.994 (0.003)**	**1.00 (0.00)**
	Individual Dataset	MNISTM (N = 10,000)	0.948 (0.007)	0.998 (0.001)
	Individual Dataset	USPS (N = 2,007)	0.954 (0.007)	0.998 (0.001)

MNIST + MNISTM + USPS 100% (N = 127,291)	All Datasets	MNIST + MNISTM + USPS (N = 22,007)	0.967 (0.006)	0.999 (0.00)
	Individual Dataset	MNIST (N = 10,000)	**0.989 (0.003)**	**1.00 (0.00)**
	Individual Dataset	MNISTM (N = 10,000)	0.954 (0.007)	0.999 (0.00)
	Individual Dataset	USPS (N = 2,007)	0.964 (0.006)	0.999 (0.00)

Supplemental Table 6. Results of baseline global models trained on incremental numbers of chest x-rays from all available hospital sites and evaluated on the global test set and site-specific test sets.

Train Site (No. Images)	Comparison Type	Test Site (No. Images)	Accuracy (SD)	AUC (SD)	Sensitivity (SD)	Specificity (SD)	PPV (SD)	NPV (SD)
Beth Israel + NIH + San Juan + Stanford 0.1% (N = 489)	All Sites	Beth Israel + NIH + San Juan + Stanford (N = 61,657)	0.703 (0.015)	**0.766 (0.015)**	0.722 (0.021)	0.686 (0.020)	0.671 (0.021)	0.736 (0.021)
	Individual Site	Beth Israel (N = 24,780)	0.677 (0.015)	0.737 (0.016)	0.675 (0.022)	0.678 (0.020)	0.616 (0.021)	0.732 (0.019)
	Individual Site	NIH (N = 11,517)	0.655 (0.015)	0.645 (0.019)	0.529 (0.032)	0.691 (0.016)	0.331 (0.026)	0.836 (0.015)
	Individual Site	San Juan (N = 5,974)	**0.797 (0.012)**	0.627 (0.023)	0.475 (0.080)	**0.809 (0.013)**	0.091 (0.020)	**0.975 (0.005)**
	Individual Site	Stanford (N = 19,386)	0.736 (0.014)	0.705 (0.018)	**0.790 (0.015)**	0.523 (0.035)	**0.866 (0.012)**	0.390 (0.030)
Beth Israel + NIH + San Juan + Stanford 1% (N = 4,891)	All Sites	Beth Israel + NIH + San Juan + Stanford (N = 61,657)	0.716 (0.015)	**0.797 (0.015)**	0.673 (0.018)	0.793 (0.022)	0.853 (0.016)	0.576 (0.023)
	Individual Site	Beth Israel (N = 24,780)	0.697 (0.015)	0.777 (0.014)	0.635 (0.019)	0.808 (0.021)	0.855 (0.016)	0.554 (0.022)
	Individual Site	NIH (N = 11,517)	0.653 (0.015)	0.676 (0.018)	0.514 (0.023)	0.747 (0.018)	0.578 (0.027)	0.694 (0.018)
	Individual Site	San Juan (N = 5,974)	0.744 (0.014)	0.704 (0.021)	0.388 (0.031)	**0.859 (0.013)**	0.474 (0.035)	**0.812 (0.014)**
	Individual Site	Stanford (N = 19,386)	**0.769 (0.014)**	0.742 (0.018)	**0.773 (0.014)**	0.727 (0.046)	**0.965 (0.007)**	0.250 (0.026)
Beth Israel + NIH + San Juan + Stanford 5% (N = 24,455)	All Sites	Beth Israel + NIH + San Juan + Stanford (N = 61,657)	0.747 (0.014)	**0.816 (0.014)**	0.764 (0.018)	0.732 (0.020)	0.725 (0.020)	0.770 (0.019)
	Individual Site	Beth Israel (N = 24,780)	0.732 (0.014)	0.797 (0.014)	0.714 (0.021)	0.749 (0.019)	0.727 (0.020)	0.737 (0.020)
	Individual Site	NIH (N = 11,517)	0.688 (0.014)	0.699 (0.017)	0.611 (0.033)	0.708 (0.015)	0.355 (0.026)	0.874 (0.014)

	Individual Site	San Juan (N = 5,974)	**0.806 (0.013)**	0.712 (0.021)	0.605 (0.081)	**0.815 (0.013)**	0.115 (0.022)	**0.981 (0.005)**
	Individual Site	Stanford (N = 19,386)	0.781 (0.013)	0.776 (0.018)	**0.828 (0.014)**	0.622 (0.033)	**0.882 (0.012)**	0.513 (0.032)
Beth Israel + NIH + San Juan + Stanford 10% (N = 48,910)	All Sites	Beth Israel + NIH + San Juan + Stanford (N = 61,657)	0.751 (0.014)	0.822 (0.013)	**0.761 (0.018)**	0.741 (0.020)	**0.741 (0.020)**	0.761 (0.019)
	Individual Site	Beth Israel (N = 24,780)	0.737 (0.014)	0.803 (0.014)	**0.717 (0.021)**	0.755 (0.019)	**0.736 (0.020)**	0.737 (0.019)
	Individual Site	NIH (N = 11,517)	0.688 (0.015)	0.704 (0.017)	**0.594 (0.031)**	0.720 (0.016)	**0.411 (0.027)**	0.843 (0.015)
	Individual Site	San Juan (N = 5,974)	0.806 (0.013)	0.711 (0.021)	**0.652 (0.098)**	0.810 (0.013)	**0.080 (0.019)**	0.989 (0.004)
	Individual Site	Stanford (N = 19,386)	0.787 (0.013)	0.788 (0.017)	**0.825 (0.014)**	0.645 (0.033)	**0.897 (0.011)**	0.496 (0.031)
Beth Israel + NIH + San Juan + Stanford 20% (N = 97,821)	All Sites	Beth Israel + NIH + San Juan + Stanford (N = 61,657)	0.752 (0.014)	0.824 (0.013)	**0.743 (0.018)**	0.762 (0.020)	**0.779 (0.019)**	0.724 (0.020)
	Individual Site	Beth Israel (N = 24,780)	0.738 (0.014)	0.807 (0.014)	**0.699 (0.020)**	0.782 (0.019)	**0.787 (0.019)**	0.693 (0.021)
	Individual Site	NIH (N = 11,517)	0.687 (0.015)	0.707 (0.017)	**0.584 (0.029)**	0.725 (0.016)	**0.439 (0.026)**	0.825 (0.015)
	Individual Site	San Juan (N = 5,974)	0.809 (0.013)	0.717 (0.021)	**0.612 (0.073)**	0.818 (0.012)	**0.139 (0.024)**	0.978 (0.005)
	Individual Site	Stanford (N = 19,386)	0.790 (0.013)	0.790 (0.017)	**0.812 (0.014)**	0.685 (0.035)	**0.925 (0.009)**	0.431 (0.030)
Beth Israel + NIH + San Juan + Stanford 40% (N = 195,643)	All Sites	Beth Israel + NIH + San Juan + Stanford (N = 61,657)	0.756 (0.014)	0.828 (0.013)	**0.746 (0.019)**	0.768 (0.019)	**0.786 (0.018)**	0.726 (0.020)
	Individual Site	Beth Israel (N = 24,780)	0.744 (0.014)	0.812 (0.014)	**0.710 (0.020)**	0.781 (0.018)	**0.780 (0.019)**	0.711 (0.020)
	Individual Site	NIH (N = 11,517)	0.694 (0.014)	0.712 (0.017)	**0.587 (0.029)**	0.738 (0.016)	**0.485 (0.026)**	0.810 (0.016)
	Individual Site	San Juan (N = 5,974)	0.814 (0.012)	0.720 (0.021)	**0.627 (0.062)**	0.826 (0.012)	**0.188 (0.028)**	0.972 (0.006)
	Individual Site	Stanford (N = 19,386)	0.792 (0.013)	0.793 (0.017)	**0.810 (0.014)**	0.696 (0.035)	**0.931 (0.009)**	0.423 (0.029)
	All Sites	Beth Israel + NIH + San Juan + Stanford (N = 61,657)	0.744 (0.014)	0.828 (0.013)	**0.707 (0.018)**	0.802 (0.020)	**0.845 (0.017)**	0.641 (0.022)
	Individual Site	Beth Israel (N = 24,780)	0.728 (0.014)	0.810 (0.014)	**0.666 (0.019)**	0.825 (0.019)	**0.857 (0.016)**	0.611 (0.022)

Beth Israel + NIH + San Juan + Stanford 60% (N = 293,465)	Individual Site	NIH (N = 11,517)	0.676 (0.015)	0.713 (0.017)	**0.543 (0.024)**	0.767 (0.017)	**0.613 (0.025)**	0.712 (0.018)
	Individual Site	San Juan (N = 5,974)	0.803 (0.013)	0.734 (0.020)	**0.525 (0.050)**	0.835 (0.012)	**0.267 (0.031)**	0.939 (0.009)
	Individual Site	Stanford (N = 19,386)	0.786 (0.013)	0.796 (0.016)	**0.794 (0.014)**	0.733 (0.039)	**0.953 (0.008)**	0.344 (0.029)
Beth Israel + NIH + San Juan + Stanford 80% (N = 391,287)	All Sites	Beth Israel + NIH + San Juan + Stanford (N = 61,657)	0.753 (0.014)	0.826 (0.013)	**0.752 (0.018)**	0.755 (0.020)	**0.765 (0.019)**	0.741 (0.020)
	Individual Site	Beth Israel (N = 24,780)	0.740 (0.014)	0.809 (0.014)	**0.701 (0.020)**	0.785 (0.019)	**0.789 (0.019)**	0.696 (0.020)
	Individual Site	NIH (N = 11,517)	0.690 (0.015)	0.708 (0.017)	**0.598 (0.031)**	0.721 (0.016)	**0.413 (0.027)**	0.845 (0.015)
	Individual Site	San Juan (N = 5,974)	0.808 (0.012)	0.731 (0.020)	**0.627 (0.082)**	0.814 (0.012)	**0.112 (0.021)**	0.983 (0.005)
	Individual Site	Stanford (N = 19,386)	0.790 (0.013)	0.795 (0.016)	**0.823 (0.014)**	0.659 (0.033)	**0.905 (0.011)**	0.485 (0.030)
Beth Israel + NIH + San Juan + Stanford 100% (N = 489,109)	All Sites	Beth Israel + NIH + San Juan + Stanford (N = 61,657)	0.757 (0.014)	0.830 (0.013)	**0.760 (0.018)**	0.754 (0.020)	**0.759 (0.019)**	0.754 (0.019)
	Individual Site	Beth Israel (N = 24,780)	0.745 (0.014)	0.815 (0.014)	**0.721 (0.020)**	0.767 (0.018)	**0.754 (0.019)**	0.736 (0.019)
	Individual Site	NIH (N = 11,517)	0.697 (0.014)	0.718 (0.017)	**0.597 (0.029)**	0.737 (0.016)	**0.476 (0.027)**	0.821 (0.015)
	Individual Site	San Juan (N = 5,974)	0.808 (0.013)	0.727 (0.021)	**0.627 (0.079)**	0.815 (0.013)	**0.116 (0.022)**	0.982 (0.005)
	Individual Site	Stanford (N = 19,386)	0.793 (0.013)	0.796 (0.017)	**0.828 (0.014)**	0.659 (0.033)	**0.902 (0.011)**	0.503 (0.031)

SUPPLEMENTAL FIGURES

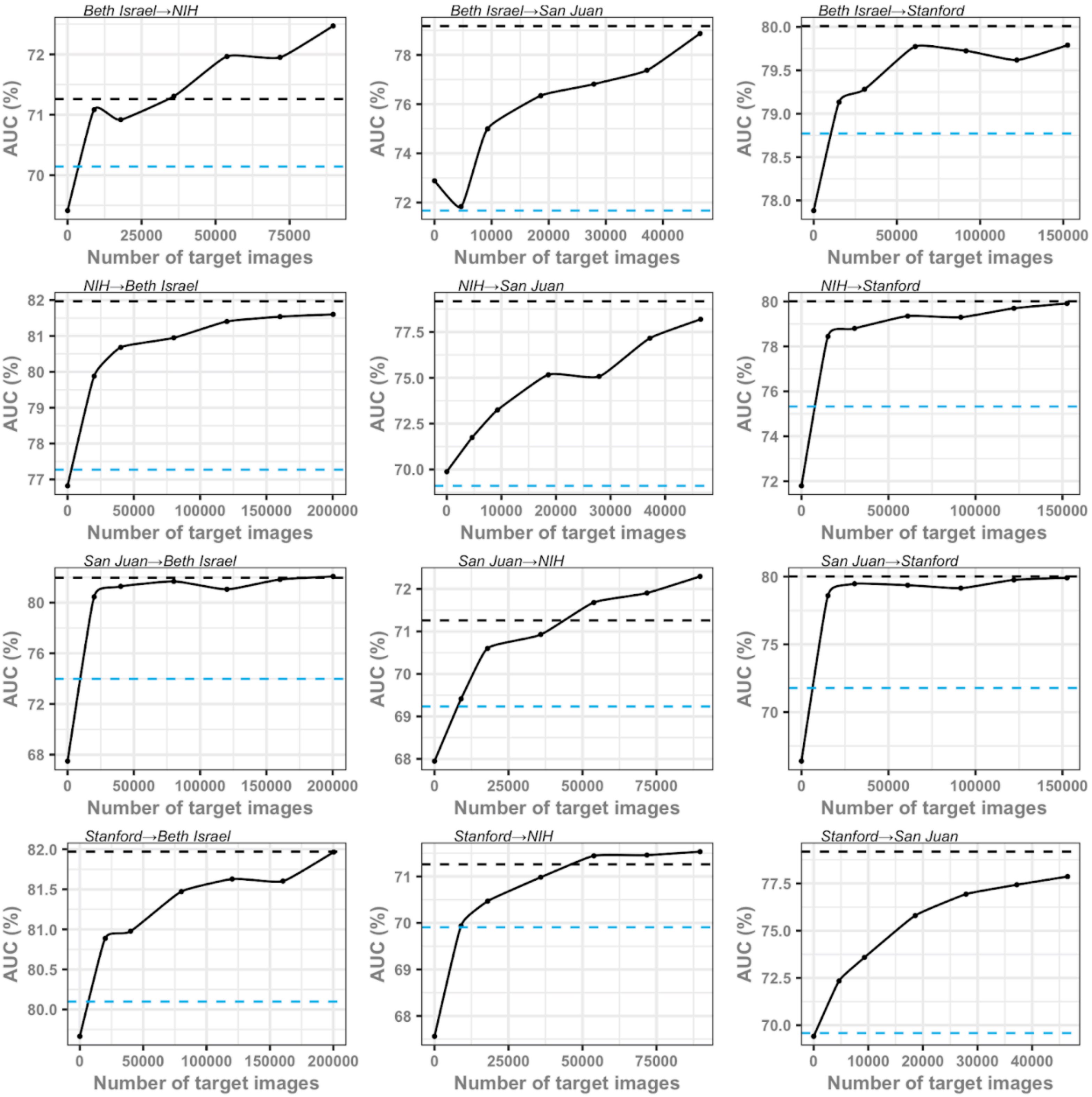

Supplemental Figure 1. Results of simulation experiments conducted to determine the number of labelled chest x-rays from the target dataset required to improve performance of the baseline classifier as measured by area under the curve (AUC). The baseline classifier trained on the source training set was fine-tuned with incremental amounts of chest x-rays from the target training set to determine gains in AUC on the target test set for each source-target dataset pair. The blue line represents performance of the adapted classifier

on the target test set whereas the black line represents the theoretical maximum performance on the target test set as obtained by a baseline classifier trained on the target training set.

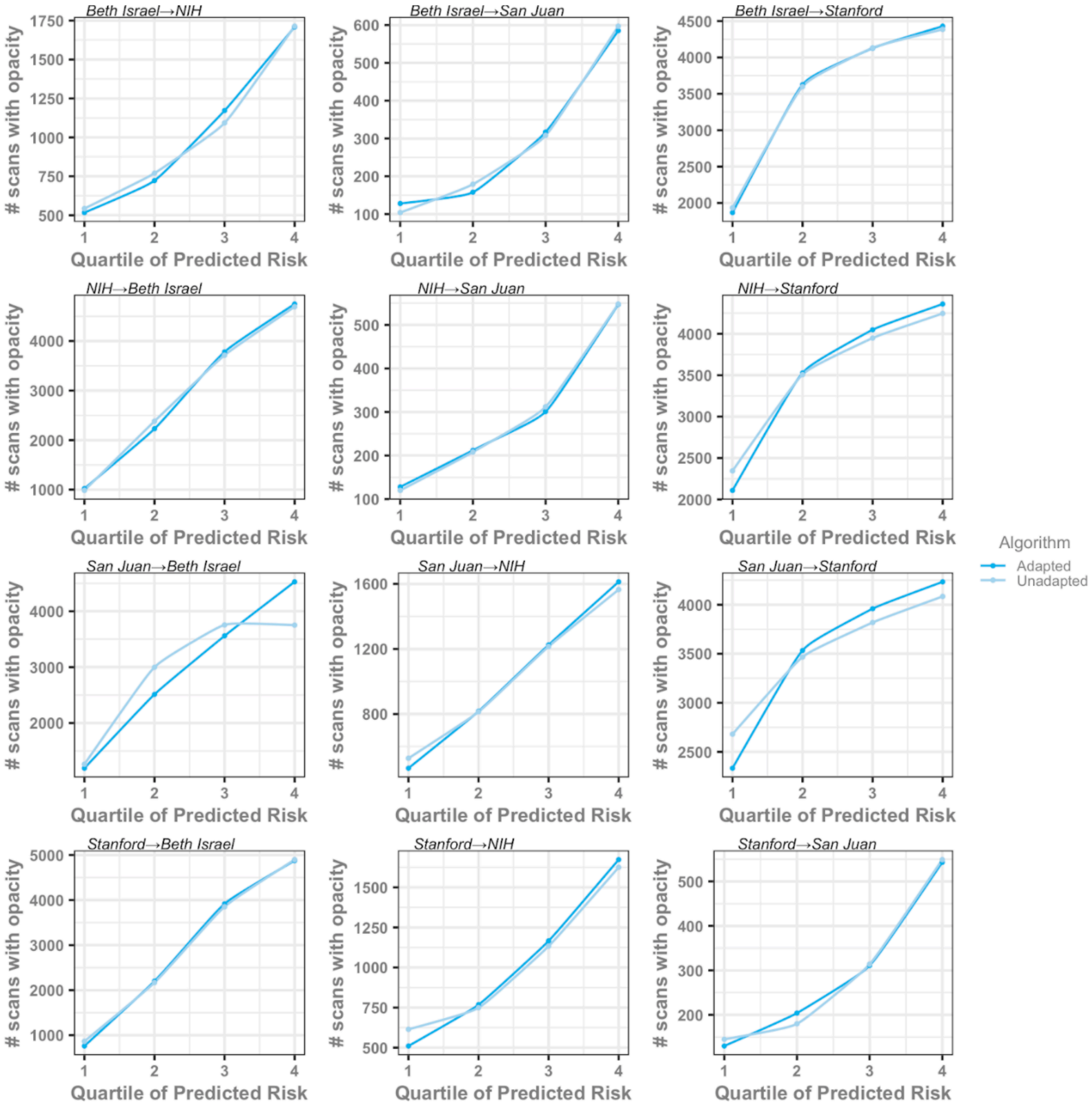

Supplemental Figure 2. Calibration curves of adapted and baseline algorithms on source-target pairs of the chest x-ray datasets.
