# Supplementary material for: Deploying deep learning models on unseen medical imaging using adversarial domain adaptation
Source: PLoS One. 2022 Oct 14;17(10):e0273262. doi: 10.1371/journal.pone.0273262 (PMC9565422; doi:10.1371/journal.pone.0273262)
Supplement: Attachment — Submitted filename: Response to Reviewers.docx [file pone.0273262.s003.docx]

30 July 2022

To Dr. Mohamed Hammad

Academic Editor, *PLOS ONE*

Thank you for your consideration of our manuscript. We have addressed all the comments provided. Below, we provide the original comments and include our point-by-point responses.

**Comments:**

**Reviewer #1:**

Since the previous version, authors have done huge work and the paper is much better. This version looks good. Therefore, I suggest accepting this paper after minor: The figures still need to be amended, where the font is too small which makes it difficult to read.

We thank the reviewer for providing comments that have improved the manuscript. To improve readability, we have provided elements of each panel as individual figures, which are now of sufficient font size to ensure all readers may view without difficulty. This will also allow the journal editing team to format the figures as they deem best in the published form of the manuscript.

**Reviewer #2:**

The authors addressed all my comments in this version of the manuscript which was well improved. I recommend it for publication.

We thank the reviewer for taking the time to provide comments that have certainly improved the manuscript.
